# Prospective participant selection and ranking to maximize actionable pharmacogenetic variants and discovery in the eMERGE Network

**DOI:** 10.1186/s13073-015-0181-z

**Published:** 2015-07-03

**Authors:** David R. Crosslin, Peggy D. Robertson, David S. Carrell, Adam S. Gordon, David S. Hanna, Amber Burt, Stephanie M. Fullerton, Aaron Scrol, James Ralston, Kathleen Leppig, Andrea Hartzler, Eric Baldwin, Mariza de Andrade, Iftikhar J. Kullo, Gerard Tromp, Kimberly F. Doheny, Marylyn D. Ritchie, Paul K. Crane, Deborah A. Nickerson, Eric B. Larson, Gail P. Jarvik

**Affiliations:** Department of Medicine, Division of Medical Genetics, University of Washington, 1705 NE Pacific Street, Seattle, 98195 WA USA; Department of Genome Sciences, University of Washington, 3720 15th Avenue NE, Seattle, 98195 WA USA; Group Health Research Institute, Group Health Cooperative, 1730 Minor Avenue, Seattle, 98101 WA USA; Department of Bioethics and Humanities, University of Washington, 1959 NE Pacific Street, Seattle, 98195 WA USA; Department of Pathology, University of Washington, 1959 NE Pacific Street, Seattle, 98195 WA USA; Division of Biomedical Statistics and Informatics, Mayo Clinic, 200 First Street SW, Rochester, 55905 MN USA; Division of Cardiovascular Diseases, Mayo Clinic, 200 First Street SW, Rochester, 55905 MN USA; The Sigfried and Janet Weis Center for Research, Geisinger Health System, 100 North Academy Avenue, Danville, 17882 PA USA; Center for Inherited Disease Research, Johns Hopkins University School of Medicine, 333 Cassell Drive, Baltimore, 21224 MD USA; Center for Systems Genomics, Department of Biochemistry and Molecular Biology, Pennsylvania State University, 512A Wartik Laboratory, University Park, 16802 PA USA; Biomedical and Translational Informatics, Geisinger Health System, 100 North Academy Avenue, Danville, 17882 PA USA; Division of General Internal Medicine, University of Washington, 325 Ninth Avenue, Seattle, 981014 WA USA

## Abstract

**Background:**

In an effort to return actionable results from variant data to electronic health records (EHRs), participants in the Electronic Medical Records and Genomics (eMERGE) Network are being sequenced with the targeted Pharmacogenomics Research Network sequence platform (PGRNseq). This cost-effective, highly-scalable, and highly-accurate platform was created to explore rare variation in 84 key pharmacogenetic genes with strong drug phenotype associations.

**Methods:**

To return Clinical Laboratory Improvement Amendments (CLIA) results to our participants at the Group Health Cooperative, we sequenced the DNA of 900 participants (61 *%* female) with non-CLIA biobanked samples. We then selected 450 of those to be re-consented, to redraw blood, and ultimately to validate CLIA variants in anticipation of returning the results to the participant and EHR. These 450 were selected using an algorithm we designed to harness data from self-reported race, diagnosis and procedure codes, medical notes, laboratory results, and variant-level bioinformatics to ensure selection of an informative sample. We annotated the multi-sample variant call format by a combination of SeattleSeq and SnpEff tools, with additional custom variables including evidence from ClinVar, OMIM, HGMD, and prior clinical associations.

**Results:**

We focused our analyses on 27 actionable genes, largely driven by the Clinical Pharmacogenetics Implementation Consortium. We derived a ranking system based on the total number of coding variants per participant (75.2±14.7), and the number of coding variants with high or moderate impact (11.5±3.9). Notably, we identified 11 stop-gained (1 *%*) and 519 missense (20 *%*) variants out of a total of 1785 in these 27 genes. Finally, we prioritized variants to be returned to the EHR with prior clinical evidence of pathogenicity or annotated as stop-gain for the following genes: *CACNA1S* and *RYR1* (malignant hyperthermia); *SCN5A*, *KCNH2*, and *RYR2* (arrhythmia); and *LDLR* (high cholesterol).

**Conclusions:**

The incorporation of genetics into the EHR for clinical decision support is a complex undertaking for many reasons including lack of prior consent for return of results, lack of biospecimens collected in a CLIA environment, and EHR integration. Our study design accounts for these hurdles and is an example of a pilot system that can be utilized before expanding to an entire health system.

**Electronic supplementary material:**

The online version of this article (doi:10.1186/s13073-015-0181-z) contains supplementary material, which is available to authorized users.

## Background

The Clinical Pharmacogenetics Implementation Consortium (CPIC), both of the Pharmacogenomics Research Network (PGRN) and Pharmacogenomics Knowledge Base (PharmGKB [1]), was formed to overcome some of the barriers to individualized medicine by providing peer-reviewed, updated, evidence-based, freely accessible guidelines for gene/drug pairs [2]. One product of this Network was the PGRN sequence platform (PGRNseq). The PGRNseq target set contains the coding regions (exons), UTRs, 2kb upstream, and 1kb downstream for 84 pharmacogenes [3]. This target also includes all SNPs on the Affymetrix DMET Plus Solution array and the Illumina ADME assay. The Affymetrix DMET Plus array is a platform that contains ∼ 2000 common variants from 231 drug metabolism and transporter genes (Web Resources). The Illumina ADME assay contains 184 biomarkers in 34 drug metabolism and transporter genes, covering > 95 *%* of the PharmaADME Core list (Web Resources).

PGRN charged its sequencing resources to develop a cost-effective, highly-scalable, and highly-accurate platform of pharmacogenetic genes. The purpose was to explore rare and known common variation in key pharmacogenetic genes with strong drug phenotype associations. The sequencing resources included the Department of Genome Sciences, University of Washington, the Genome Institute at Washington University, and the Human Genome Sequencing Center at Baylor College of Medicine. Through nomination and multiple rounds of balloting, the final consensus list included 84 pharmacogenes. The final list of genes included three classes: 1. Drug-metabolizing enzymes; 2. Drug transporters; and 3. Drug targets. While many of the genes were deemed clinically actionable by CPIC [4], some genes had little known beyond strong preliminary association data to pharmacological traits [3]. To aid in the design and accuracy testing of the target, 96 samples (32 trios) of diverse ancestry were utilized through comparisons of orthogonal data sets, duplicates across resources, and Mendelian inconsistencies. In general, there was > 99.0 *%* concordance for these controls using multiple comparison approaches [3].

Approximately 9000 participants in the Electronic Medical Records and Genomics (eMERGE) Network are currently being sequenced with PGRNseq. The eMERGE Network comprises seven adult, and three pediatric United States (US) sites with biobanks linked to electronic health records (EHRs), sponsored by the National Human Genome Research Institute (NHGRI) [5, 6]. The main focus of the NHGRI for this project was to provide eMERGE participants with the PGRNseq platform in anticipation that Clinical Laboratory Improvement Amendments (CLIA) [7]-validated actionable results would be returned to the participant and the EHR, and to characterize novel variants [8].

Our study design at the Group Health Research Institute (GH) was different from most other eMERGE sites. Most sites’ biobanks, like ours, lacked CLIA compliant samples and/or consent to return genetic results and needed to resample and/or consent participants. In our case, rather than redrawing all participants in a CLIA laboratory prior to running the PRGNSeq, we found it more efficient to sequence 900 existing non-CLIA samples from ∼ 6300 eligible biobanked participants at GH, and then recollect 450 participants of interest. As such, our goal was to prioritize our 900 sequenced participants based on potential impact of actionable results to help make choices around re-sampling and re-consenting. Here we describe the algorithm we developed to select participants with the greatest potential for actionable variants (the “selection algorithm,”) and the algorithm we developed to rank variants with highest impact (the “ranking algorithm”). The selection algorithm was designed to enrich for participants of non-European ancestry with conditions likely to be due to variants in the pharmacogenetic (PGx) genes that the ranking algorithm identified as most likely to be clinically actionable. The system we developed to deploy these algorithms will serve as a foundation for identification of potentially actionable variants and EHR integration. These data will inform pathogenicity of specific variants and practices for EHR integration of genomic data.

## Methods

### Participant selection algorithm

Potential GH participants for the PGx project were enrolled in the eMERGE Network through the Northwest Institute of Genetic Medicine (NWIGM) biorepository, and provided the appropriate consent to receive clinically relevant genetic results (*N*∼6300). Participants were eligible if aged 50−65 years old at the time of their enrollment into the NWIGM repository, living, enrolled in GH’s integrated group practice, and had completed an online health risk appraisal. This age range provided a viable target range for medication use. The selection algorithm was based on several data sources from the EHR at GH (Additional file 1: Figure S1): 1. Demographics - participants with self-reported race as Asian or African ancestry were prioritized and selected to enrich for non-European ancestry genetic variation; 2. Diagnosis and procedure codes - participants were selected if found to have EHR evidence of malignant hyperthermia and long QT syndrome (LQTS) to enrich for phenotypes related to PGx decision support. To enrich for phenotypes that could require medications for PGRNSeq drug targets, participants were selected if found to have EHR evidence of atrial fibrillation (AF), arrhythmia, congestive heart failure (CHF), or hypertension; 3. Laboratory values - if a participant had any laboratory event of creatine kinase (CK) > 1000, and were dispensed statins within 6 months of the event, then they were selected. High levels of CK could indicate a statin-related myopathy; and 4. Medications - participants were excluded if ever prescribed carbamazepine or had a current regimen of warfarin. Participants using carbamazepine likely would have been tested for genotypes in *HLA-B* prior to this study or have known tolerance to the drug. Pharmacogenetic variants in *CYP2C9* and *VKORC1* only affect starting dose for participants prescribed warfarin, thus patients already on warfarin would not benefit from these results.

### Sequencing, variant calling and annotation

We sequenced 600 participants at the University of Washington (UW), and sequenced 300 at the Center for Inherited Disease Research (CIDR). There were 894 sequence data sets that passed quality control, and we included the BAM files in multisample variant calling using the Genome Analysis Toolkit (GATK, version 2.6-5-gba531bd) with target = PGX [9–11]. The genome reference utilized was assembly BWA 0.7.4/Homo sapiens assembly19.fasta, and dbSNP137.vcf build. The annotation was standard in discovery mode, emitting variants only, using the GLM model for SNP + INDEL. We used a minimum base quality allowed of 25. Initially, we annotated the multisample VCF with the SnpEff genetic variant annotation and effect prediction toolbox [12]. Next, we annotated the 894 participant multisample.vcf with SeattleSeq (Web Resources), with additional custom variables including evidence from ClinVar [13], OMIM [14], and HGMD [15] with hyper-links to prior clinical associations.

### Participant ranking algorithm

We next ranked the 894 participants based on potential impact of actionable results (Additional file 2: Figure S2). Our goal was to identify a subset of this group to target for re-consent, blood redraw, and CLIA validation of variants in anticipation of returning results to the EHR. Our analytic pipeline included participant-level variant indexing, custom annotation, and R and LATE X scripts. It soon became apparent that we needed a relational data base model to organize the data for the analysis presented.

We created separate tables for participant-level and variant-level data, illustrated in Additional file 3: Figure S3. To join the two, we created a gene index variable, which corresponds to the genotype columns in the participant-level data, and an index variable in the variant-level table. These indices provide meta-data for the given variant, which allows for quick extraction of information. Using the example chr1.pos237754201.refG.altper0.11.geneRYR2 we know the following about this variant: 1. The variant is found on chromosome 1; 2. The position on chromosome 1 is 237754201; 3. The nucleotide reference for this allele is guanine. 4. The alternative allele frequency is 0.11 *%*; and 5. This variant is found in gene *RYR2*. In the participant level table, IUPAC notation [16] was utilized to represent genotypes in a single column (Additional file 4: Table S1).

In order to enrich for non-European ancestry and actionable indications in the 894 sequenced participants, we selected all non-Europeans using self-identified race and all with a diagnosis of long QT syndrome. To rank the remaining participants based on variants, we focused our analyses on 27 genes (Table 2) deemed either as actionable by CPIC [4], or as important drug targets based on preliminary association data to pharmacological traits [3]. We generated three variant-level variables to rank the impact.

The “total variants” and “coding variants” contain the overall number of variants (minor allele) for a given participant selected for the 27 genes (Additional file 4: Table S2), and for coding variants annotated as having high/moderate impact according to SnpEff, respectively. Next, we prioritized variants to be returned to the EHR at GH with prior clinical evidence of pathogenicity or annotated as stop-gain for the following genes: 1. *CACNA1S* and *RYR1* (malignant hyperthermia); 2. *SCN5A*, *KCNH2*, and *RYR2* (arrhythmia); and 3. *LDLR* (high cholesterol), as “gh variants”. We then ranked the participant list by “gh variants”, “coding variants”, and “total variants” to create an overall ranking beyond the participants already selected because of ancestry and actionable indications.

Finally, using laboratory data we created flags (1 or 0) to indicate high median laboratory values based on repeated measures for participants. The laboratory values chosen could indicate important biological events. The labs of interest included low-density lipoprotein (LDL) and triglycerides because of the lipid trait genes found on the target, including *LDLR*. High levels of CK could indicate a statin-related myopathy, among other conditions. High levels of thyroid stimulating hormone (TSH) could indicate risk for hypothyroidism or suggest an altered rate of drug metabolism [17]. We included international normalized ratio (INR) because of the risk of bleeding for participants on anticoagulant therapy and/or the presence of liver disease. We addressed multiple values for each participant by considering each person’s median value for each laboratory assay. We flagged participants if median vales of repeated measures met the following threshold: 1. LDL > 155 $\frac {mg}{dL}$; 2. Triglyceride > 288 $\frac {mg}{dL}$; 3. CK > 174 $\frac {IU}{L}$; 4. TSH > 4 $\frac {\mu g}{dL}$; and 5. INR > 1.5. These thresholds were chosen based on the 90^*th*^ percentile of observed distributions (data not shown). We created an overall sum of the five laboratory flags (1 or 0), and treated the variable as an element in our ranking algorithm.

### Research conformity to the Helsinki Declaration

GH and the UW are institutions engaged in human subjects research that have each obtained Federal Wide Assurance of Compliance (GH: FWA 00002669; UW: FWA 00006878) approved by the Office for Human Research Protection (OHRP). The FWA is a binding written agreement that research is guided by statements of principles to protect the rights and welfare of human subjects research conducted by these institutions. The statement of principles include observance with appropriate existing codes in the Declaration of Helsinki, adherence of ethical standards stated in the Belmont Report and full compliance with the Code of Federal Regulations Title 45 Part 46. All research activities were reviewed and approved by GHC’s institutional review board (IRB), the Group Health Human Subjects Review Committee, and all research subjects engaged in the informed consenting process.

NWIGM participants were informed that their DNA could be used for future research, which included the possibility of discovering a medical condition or disease not previously known. The initial NWIGM samples were not CLIA compliant, so all participants that we deemed as having a high potential impact for actionable results, and that agreed to the future research question that could affect medical care, were contacted for re-consent. We did not specifically indicate there were any “interesting” sequencing results.

### Data deposition

These data will be made available to the public through two resources. The raw data will be deposited in dbGaP, as both individual BAM files and as a multisample variant call format (VCF) file (accession #: phs000906.v1.p1). The data will also be available through aggregate forms in the **S**equence, **P**henotype, and P**h**armacogenomics **In**tegration E**x**change (SPHINX) portal (Web Resources). SPHINX contains secure, deidentified, Web-accessible repository of genomic variants, searchable by gene, pathway, and drug [8].

## Results and discussion

### Participants

We present summary statistics of demographic data by self-reported sex for the eMERGE participants are found in Table 1. Roughly 61 % of the 894 participants are female. While we enriched for non-European ancestry, 84 % of the participants self-identified as white. This is a lower value than the makeup of GH as a whole, which is about 98 % European ancestry. The next largest racial group, 8 %, self-identified as Asian. Other groups represented self-identified as Black or African American (5 %,) American Indian or Alaska Native (1 %,) Native Hawaiian or other Pacific Islander (<1 %,) and Unknown (2 %). For this sample, 3 % of our participants self-identified as Hispanic. All subjects self-identified as non-European ancestry (*N*=123) were selected to be re-consented as the highest priority.

**Table 1:** Summary statistics of demographic data for the eMERGE participants with PGRNSeq data by self-reported sex and race

	Female	Male	Combined
	(*N*=546)	(*N*=348)	(*N*=894)
Self-Reported Race			
American Indian orAlaska Native	2 *%*(9)	1 *%*(2)	1 *%*(11)
Asian	9 *%*(50)	5 *%*(19)	8 *%*(69)
Black or African American	5 *%*(29)	3 *%*(12)	5 *%*(41)
Native Hawaiian or otherPacific Islander	0 *%*(2)	0 *%*(0)	0 *%*(2)
Unknown	1 *%*(4)	5 *%*(16)	2 *%*(20)
White	83 *%*(452)	86 *%*(299)	84 *%*(751)
Ethnicity			
No	95 *%*(520)	94 *%*(326)	95 *%*(846)
Yes	4 *%*(24)	2 *%*(6)	3 *%*(30)
Unknown	0 *%*(2)	5 *%*(16)	2 *%*(18)
Hx ^*a*^ of LQTS	0 *%*(0)	1 *%*(2)	0 *%*(2)
Hx of Hypertension	79 *%*(429)	80 *%*(279)	79 *%*(708)
Hx of Arrhythmia	48 *%*(264)	53 *%*(186)	50 *%*(450)
Hx of AF	7 *%*(38)	14 *%*(47)	10 *%*(85)
Hx of CHF	1 *%*(6)	3 *%*(12)	2 *%*(18)
Median LDL $> 155\frac {mg}{dL}$	9 *%*(50)	6 *%*(22)	8 *%*(72)
Median Triglyceride$> 22\frac {mg}{dL}$	6 *%*(33)	11 *%*(40)	8 *%*(73)
Median CK $ > 174\frac {IU}{L}$	1 *%*(4)	7 *%*(23)	3 *%*(27)
Median TSH $> 4\frac {\mu g}{dL}$	6 *%*(34)	4 *%*(13)	5 *%*(47)
Median INR >1.5	7 *%*(36)	11 *%*(40)	9 *%*(76)
Total PGx Variants(total variants)	75.8±15.9	74.3±12.8	75.2±14.7
Coding PGx Variants(coding variants)	11.6±4.2	11.4±3.5	11.5±3.9
Group Health PGx Variants (gh variants)	40.0±9.6	38.5±8.7	39.4±9.3

The continuous variables total variants, coding variants, and gh variants are presented in terms of mean and ± standard deviation

^a^Hx = History found in the EHR

Next, we mined the EHR and flagged the subset of the 894 participants with usable PGRNSeq data with diagnosis or procedures codes that could provide actionable indications related to PGRNSeq genes (Table 1). Two participants had a history of LQTS, and were selected on that basis in the ranking algorithm. Roughly 80 *%* of male and female participants had a diagnosis or procedure code related to hypertension. This proportion was found in both males and females. Roughly 50 *%* of the participants had a diagnosis or procedure code related to arrhythmia. Again, this approximate proportion was found in both sex groups. We observed a difference in the sexes with respect to history of atrial fibrillation. More males had a history of AF (14 %) compared to females (7 %). Only 2 % of the participants had a history of CHF. Next, we flagged participants with five median laboratory values that could indicate medical conditions that may require medication, including high lipids, hypothyroidism, and being anticoagulated. The laboratory values included LDL, triglyceride, CK, TSH, and INR (Table 1). For the most part, the proportion of subjects flagged for each category was <10 *%* with moderate differences between the groups. The sum of all these laboratory values produced the weighted laboratory variable. The males had a mean of 0.397, compared to the female’s 0.288 (data not shown). The three numbers for “total variants,” “coding variants,” and “gh variants” represent quartiles of the distributions (25^*th*^,50^*th*^, and 75^*th*^). Both groups had an approximate median of 74 variants, and an approximate median of 11 coding variants with high or moderate impact. We used these variables along with “gh variants” variable to rank the participants.

**Fig. 1:**
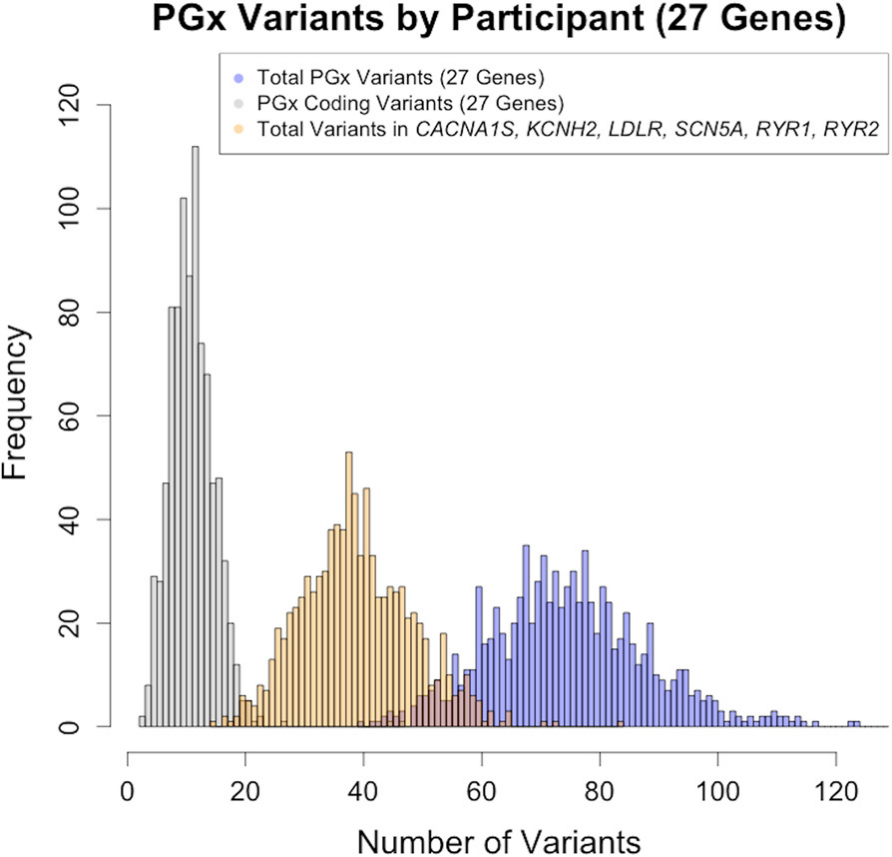
PGx Variants by Participants (27 Genes). Distributions of total variants, high-moderate impact coding variants, and total variants for the following genes: 1. *CACNA1S* and *RYR1* (malignant hyperthermia); 2. *SCN5A*, *KCNH2*, and *RYR2* (arrhythmia); and 3. *LDLR* (high cholesterol)

**Fig. 2:**
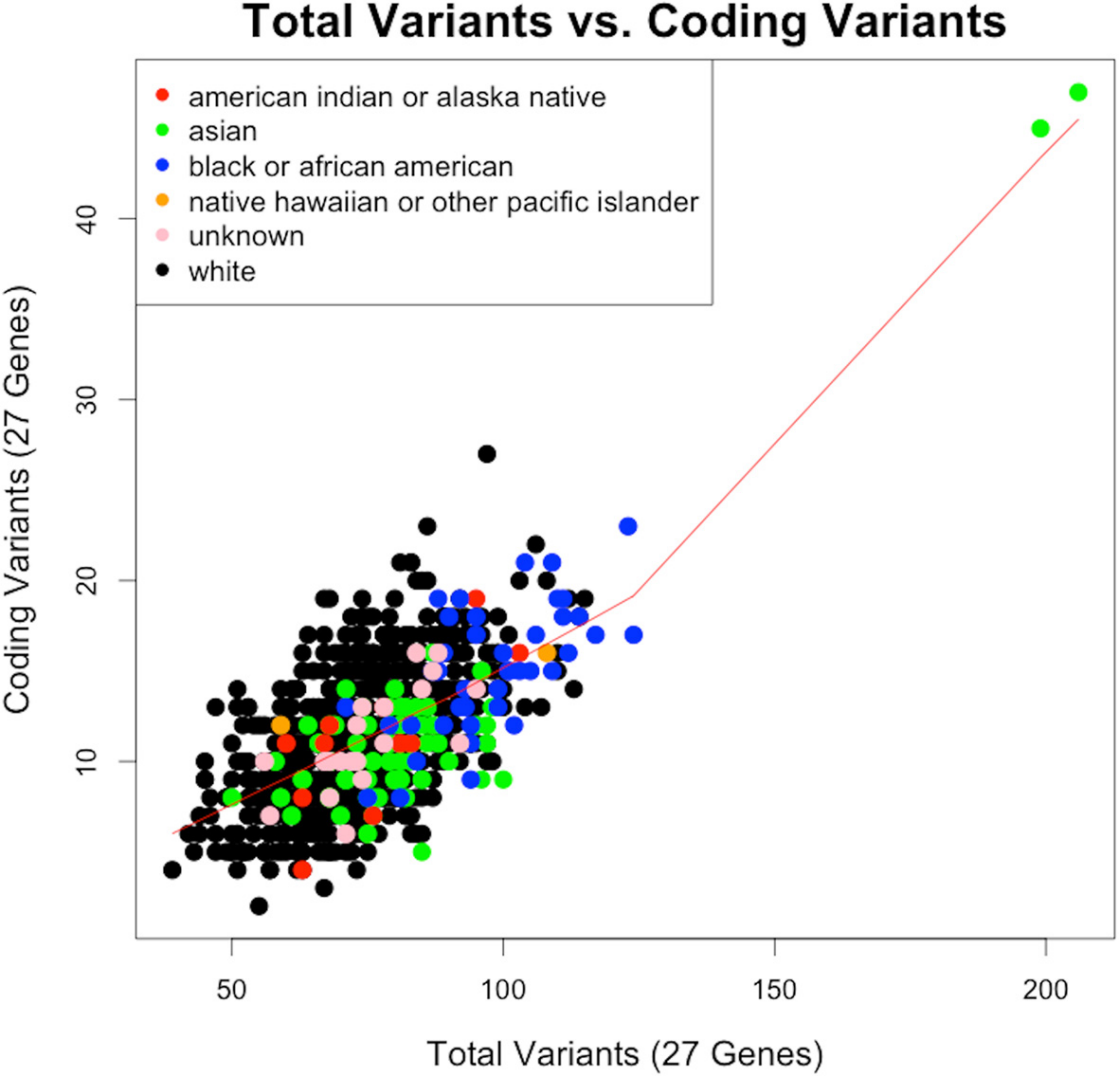
Total Variants vs. Coding Variants. Total number of variants versus the number of high/moderate impact coding variants by participant. The 27 genes are listed in Additional file 4: Table S2, and the impact assignment is according to SnpEff annotation tool

For the sample of 894 participants, we identified a total of 1785 variants in the 27 genes. Overall, the number of prioritized variants for return of results at GH (mean = 39.4; standard deviation =±9.3), the total number of variants per participant (75.2±14.7), and the number of coding variants with high or moderate impact (11.5±3.9) did not significantly differ between males and female. These three variables, prioritized, total, and impact variants, in the respective order presented were used to prioritize the participants who had not already been selected on the basis of having non-European ancestry or a history of LQTS. All three distributions are summarized in Fig. 1. All three variables approximate a normal distribution, but there are two outliers each due to an excess of variants. The number of coding variants correlates with high or moderate impact variants as illustrated in Fig. 2. The plot illustrates the correlation using a Lowess smoothing function, and is annotated by self-reported race. The two outlier individuals are self-identified as Asian.

### Variants identified

Table 2 shows summary statistics of variant-level data for the eMERGE participants by annotated impact, whether high/moderate coding or other. The complete list of variant effect prediction details can be found on the SnpEff web site (Web Resources). In general, single nucleotide variants, structural variation, and copy number variation are labeled as having high or moderate impact if annotated as modifying the coding and/or splice regions of a given gene.

**Table 2:** Summary statistics of variant-level data for the eMERGE participants by effect prediction, whether coding with high/moderate impact and lower impact

Impact	Other	High/Moderate	Combined
	(*N*=1253)	(*N*=532)	(*N*=1785)
Variant Function			
3-prime-UTR	26 *%*(328)	0 *%*(0)	18 *%*(328)
5-prime-UTR	6 *%*(71)	0 *%*(0)	4 *%*(71)
intron	30 *%*(377)	0 *%*(0)	21 *%*(377)
intron-near-splice	2 *%*(19)	0 *%*(0)	1 *%*(19)
missense	0 *%*(3)	95 *%*(506)	29 *%*(509)
missense-near-splice	0 *%*(0)	2 *%*(10)	1 *%*(10)
non-coding-exon	0 *%*(3)	0 *%*(0)	0 *%*(3)
splice-acceptor	0 *%*(0)	1 *%*(3)	0 *%*(3)
splice-donor	0 *%*(0)	0 *%*(2)	0 *%*(2)
stop-gained	0 *%*(0)	2 *%*(11)	1 *%*(11)
synonymous	36 *%*(451)	0 *%*(0)	25 *%*(451)
synonymous-near-splice	0 *%*(1)	0 *%*(0)	0 *%*(1)
Prior rsID	67 *%*(836)	71 *%*(376)	68 *%*(1212)
Prior Clinical Association	6 *%*(72)	18 *%*(96)	9 *%*(168)
OMIM	0 *%*(6)	1 *%*(3)	1 *%*(9)
ClinVar	1 *%*(12)	3 *%*(17)	2 *%*(29)
HGMD	2 *%*(23)	30 *%*(157)	10 *%*(180)
Gene List			
*ABCA1*	9 *%*(114)	8 *%*(44)	9 *%*(158)
*ABCB1*	5 *%*(63)	6 *%*(32)	5 *%*(95)
*APOA1*	0 *%*(4)	1 *%*(6)	1 *%*(10)
*CACNA1S*	4 *%*(55)	9 *%*(46)	6 *%*(101)
*CYP2C19*	2 *%*(29)	3 *%*(18)	3 *%*(47)
*CYP2C9*	4 *%*(45)	4 *%*(19)	4 *%*(64)
*CYP2D6*	11 *%*(139)	10 *%*(54)	11 *%*(193)
*CYP3A4*	4 *%*(45)	2 *%*(8)	3 *%*(53)
*CYP3A5*	0 *%*(3)	0 *%*(0)	0 *%*(3)
*DPYD*	4 *%*(50)	5 *%*(24)	4 *%*(74)
*HMGCR*	5 *%*(59)	1 *%*(6)	4 *%*(65)
*KCNH2*	7 *%*(82)	4 *%*(19)	6 *%*(101)
*LDLR*	7 *%*(84)	3 *%*(16)	6 *%*(100)
*NAT2*	1 *%*(8)	2 *%*(13)	1 *%*(21)
*RYR1*	11 *%*(139)	19 *%*(102)	14 *%*(241)
*RYR2*	12 *%*(149)	8 *%*(41)	11 *%*(190)
*SCN5A*	8 *%*(98)	8 *%*(45)	8 *%*(143)
*SLCO1B1*	2 *%*(20)	5 *%*(25)	3 *%*(45)
*TPMT*	3 *%*(40)	2 *%*(8)	3 *%*(48)
*VKORC1*	2 *%*(27)	1 *%*(6)	2 *%*(33)
Average Depth	295/401/473	347/436/499	308/413/480
GERP	−3.44/−0.47/1.61	2.01/3.90/4.82	−2.31/0.49/3.50
CADD	2.12/5.37/8.66	10.34/14.36/18.59	3.10/7.28/11.70

Effect prediction details can be found on the SnpEff web site (Web Resources). In general, single nucleotide variants, structural variation, and copy number variation annotated as having high or moderate impact modify the coding and/or splice regions of a given gene. For the continuous variables average depth, GERP, and CADD, the three numbers represent quartiles of the distributions (25^*th*^,50^*th*^, and 75^*th*^)

Of the 532 coding variants identified to have high or moderate impact in our sample, 95 *%* (506) were labeled as missense, 2 *%* (10) as missense-near-splice, 1 *%* (3) as splice-acceptor, <1 *%* (2) as splice-donor, and 2 *%* (11) as stop-gained. Most of the 1253 variants not annotated as having a coding with high/moderate effect were synonymous (*N*=451; 36 *%*), followed by 3-prime-UTR (*N*=328; 26 *%*), and 5-prime-UTR, (*N*=71; 6 *%*). There were also variants annotated as intron-near-splice, missense, non-coding-exon, and synonymous-near-splice.

For average depth (across all participants), the CADD score [18], and the GERP score [19], the three numbers represent quartiles of the distributions (25^*th*^,50^*th*^, and 75^*th*^). The average read depth was greater than 400 for both the coding variants with moderate/high impact as well as other classes of variants. The median GERP score for the high/moderate group was 3.9, compared to the other variants at -0.47. The CADD score, which is correlated with the GERP, had a median value of 14.36 in the high/moderate group, compared to the other variants at 5.37.

Roughly 30 *%* of the variants for both the coding with high/moderate impact and lower impact had not been assigned an rsID. Based on annotation programs, 18 *%* (*N*=96) of the 543 high/moderate impact variants had a prior clinical association, while 6 *%* (*N*=72) of the 1253 lower impact variants had one. Less than 1 % of the variants were found in OMIM for both high/moderate and lower impact classes. ClinVar annotation was found for 3 % (*N*=17) of the coding high/moderate variants and 1 % (*N* = 12) of the other variants. We observed a major difference in the classes for the HGMD annotation. Thirty percent (*N*=157) of the coding high/moderate variants had HGMD annotation vs. 2 *%* (*N*=23) for the other variants.

We have provided the list of genes with variants sub-classified by each annotated effect prediction class (Table 2). As expected, not all genes are represented in this list, most notably *HLA-B*. The large gene *RYR1* had the most variants 14 *%* (*N*=241) of total variants identified in our sample, and 19 *%* (*N*=102) of the 532 variants in the coding high/moderate class. *CYP2D6* had 11 *%* (*N*=193) of the total variants, with similar results in both high/moderate and lower impact groups, followed by *RYR2* at 11 % (*N*=190) of total variants, and 8 *%* (41) of the high/moderate group.

In addition to the algorithm outlined, we identified variants on a participant level we deemed important for re-consenting. We identified 20 participants with variants having ClinVar annotation as pathogenic or likely pathogenic. Next, we identified 24 participants with variants annotated as stop-gained according to SnpEff. We used HGMD annotation to identify 133 participants with putative disease-causing or frameshift/truncating variants. Finally, we identified 134 participants with prior clinical evidence as pathogenic or likely pathogenic reviewed by experts in our Exome Variant Server 6500 participant project [20]. Many of these variants overlapped for a given participant, resulting in 134 unique participants identified through these tools.

We identified 516 coding, 11 stop-gained, and 5 splice acceptor/donor variants in 27 pharmacogenes in our sample of 894 participants. For the 20 out of 27 genes listed in Table 2 with variants identified, there was an average of 26.6 coding and/or splice variants per gene. Approximately 70 % of these had rsIDs, most likely because of the inclusion of SNPs from the Affymetrix DMET Plus Solution array and Illumina ADME assay on the PGRNseq platform. Eighteen percent of the 532 variants annotated as having high or moderate impact had a prior clinical association through HGMD for 15 of the 20 genes listed in Table 2. Five of the twenty genes did not have variants annotated as having high/moderate impact.

The majority of these variants (33 % of variants with high/moderate impact with a prior clinical association) fall into the sodium channel, voltage-gated, type V, alpha subunit (*SCN5A*) gene. This gene contains potential actionable variants for arrhythmia because it codes for sodium channels for cardiac electrical signal transmission. The next two major genes with large numbers of coding variants were ryanodine receptor 1 (*RYR1*) at 18 %, and potassium voltage-gated channel, subfamily H (eag-related), member 2 (*KCNH2*) at (11 %). If pathogenic, variants found in *RYR1* indicate clinical actionability for malignant hyperthermia, and variants found in *KCNH2* present actionability for arrhythmia similar to *SCN5A*. The high number of novel variants found suggests the need to classify the pathogenicity of these variants in order for clinical sequencing to be most useful.

The analytic pipeline we developed for this project, including participant-level variant indexing, custom annotation, and R and LATE X scripts, will serve as a foundation for identification of potentially actionable variants and EHR integration for our site. These data will inform pathogenicity of specific variants and practices for EHR integration of genomic data for clinical decision support (CDS) activities.

The strategy employed here relied on a high confidence that participants who had non-CLIA PGRNSeq tests would return to provide samples for CLIA testing. Within two months of beginning re-consent, 450 of 529 (85 *%*) participants contacted provided consent and blood samples for Phase II, and validated results are already being returned to participants. As approved by our IRB, we specifically included a significant proportion of participants with no interesting variants in the recontact so that the fact of being recontacted did not indicate that there was an interesting variants. Participants were not given any indication of whether we had a suggestion of results of interest or not.

## Conclusions

NHGRI’s implementation of the PGRNseq target in 9000 participants in the eMERGE Network and subsequent implementation into the EHR will be a milestone in the quest for personalized medicine as it advances the national electronic health information infrastructure. This project provided us the unique opportunity to holistically maximize actionable variants to return to 450 of 894 participants through the EHR based on both phenotype data derived from the EHR and sequence data.

Given our study design at GH, we chose to sequence 900 of our non-CLIA samples from ∼ 6300 eligible biobanked participants, and then recollect 450 participants of interest for CLIA validation. We felt this process was more efficient than re-consenting all eligible biobanked participants. This approach enabled us to gain experience in the selection and ranking of participants based on potential impact of clinically actionable PGx results to return to the EHR.

Our approach did have limitations. While we were interested in and over-selected for non-European participants, that did not necessarily translate into a greater potential for clinically actionable PGx variants. By sequencing first and ranking, we placed great confidence that the GH participants would return to provide blood samples for CLIA testing. This approach relied heavily on a motivated cohort, and may not be appropriate for all health system cohorts if not the case. Prospectively enrolling participants, as was the study design for many other eMERGE sites participating in this project, would have provided more precision in identifying actionable results to be returned to the participant and the EHR. Multiple eMERGE sites used a predictive algorithm to estimate risk of of receiving drugs like simvastatin, clopidogrel, or warfarin [8]. The selection of our list of 27 genes from the platform could be considered subjective. We started with a list of actionable variants identified by CPIC, and added disease genes of interest for our cohort. Finally, while mining participant data from EHRs does provide excellent potential for phenotyping, there are limitations to its granularity.

The incorporation of genetics into the EHR for CDS is a complex undertaking for many reasons including lack of prior consent for return of results, lack of biospecimens collected in a CLIA environment, and EHR interfacing and integration. Many institutions will have to pilot systems such as the one presented in this manuscript to understand and account for these hurdles before expanding to an entire health system population.

Variants found in PGx genes are obvious candidates for CDS activities. As biotechnology advances to identify new genomic variation and the field of bioinformatics advances to identify novel function, the research area of genomic integration into the EHR for CDS will become more important.

The PGRNSeq data will be the basis for PGx studies in the eMERGE network, and will lead ultimately to clinical implementation. We plan to identify variants associated with medication adverse events and efficacy to determine associated variants. In addition, we are analyzing lipid traits for ∼9000 eMERGE participants based on candidate genes, including *LDLR*, for single and multiple variant gene-based association discovery work.

## Web resources

- ClinVar: http://www.ncbi.nlm.nih.gov/clinvar/
- CPIC: http://www.pharmgkb.org/page/cpic
- dbGaP: http://www.ncbi.nlm.nih.gov/gap
- DMET+: http://www.affymetrix.com/catalog/131412/AFFY/DMET-Plus-Solution#1_1
- HGMD: http://www.hgmd.cf.ac.uk/ac/index.php
- Group Health Research Institute (GHRI): http://www.grouphealthresearch.org/
- Illumina ADME: http://support.illumina.com/array/array_kits/veracode_adme_core_panel/documentation.html
- LATE X: http://www.latex-project.org/
- OMIM: http://www.ncbi.nlm.nih.gov/omim
- PharmaADME.org: http://www.pharmaadme.org/joomla/
- Pharmacogenomics Research Network (PGRN): http://pgrn.org/display/pgrnwebsite/PGRN+Home
- PharmGKB: https://www.pharmgkb.org/
- R Statistical Computing: http://www.r-project.org/
- R Hmisc library: http://cran.r-project.org/web/packages/Hmisc/index.html
- SeattleSeq Annotation: http://snp.gs.washington.edu/SeattleSeqAnnotation138
- SnpEff: Genetic variant annotation and effect prediction toolbox: http://snpeff.sourceforge.net/index.html
- SPHINX: https://www.emergesphinx.org/
- SQLite Database: https://sqlite.org/

## Additional files

Additional file 1
**Figure S1**. Selection algorithm for the prospective participant selection and ranking to maximize actionable pharmacogenetic variants and discovery in the eMERGE Network. (PDF 43.9KB)

Additional file 2
**Figure S2.** Ranking algorithm for the prospective participant selection and ranking to maximize actionable pharmacogenetic variants and discovery in the eMERGE Network. (PDF 73.0KB)

Additional file 3
**Figure S3.** System desgin for the prospective participant selection and ranking to maximize actionable pharmacogenetic variants and discovery in the eMERGE Network; *†*=IUPAC notation. (PDF 94.5KB)

Additional file 4
**Table S1.** IUPAC Ambiguity Codes; **Table S2.** PGx Gene List. (PDF 74.9KB)

## Abbreviations

AF: Atrial fibrillation
CDS: Clinical decision support
CHF: Congestive heart failure
CIDR: Center for Inherited Disease Research
CK: Creatine kinase
CLIA: Clinical Laboratory Improvement Amendments
CPIC: Clinical Pharmacogenetics Implementation Consortium
EHR: Electronic health records
eMERGE Network: Electronic Medical Records and Genomics
GATK: Genome Analysis Toolkit
GH: Group Health Research Institute
HGMD: Human Gene Mutation Database
INR: International normalized ratio
IRB: Institutional review board
kb: Kilobase
LQTS: Long QT syndrome
LDL: Low-density lipoprotein
NWIGM: Northwest Institute of Genetic Medicine
OMIM: Online Mendelian Inheritance in Man
PGRNseq: PGRN sequence platform
PGRN: Pharmacogenomics Research Network
SPHINX: Sequence, Phenotype, and Pharmacogenomics Integration Exchange
TSH: Thyroid stimulating hormone
UW: University of Washington
