# Supplementary material for: Prospective participant selection and ranking to maximize actionable pharmacogenetic variants and discovery in the eMERGE Network
Source: Genome Med. 2015 Jul 3;7(1):67. doi: 10.1186/s13073-015-0181-z (PMC4517371; doi:10.1186/s13073-015-0181-z)
Supplement: Additional file 1 — Figure S1. Selection algorithm for the prospective participant selection and ranking to maximize actionable pharmacogenetic variants and discovery in the eMERGE Network. (PDF 43.9KB) [file 13073_2015_181_MOESM1_ESM.pdf]

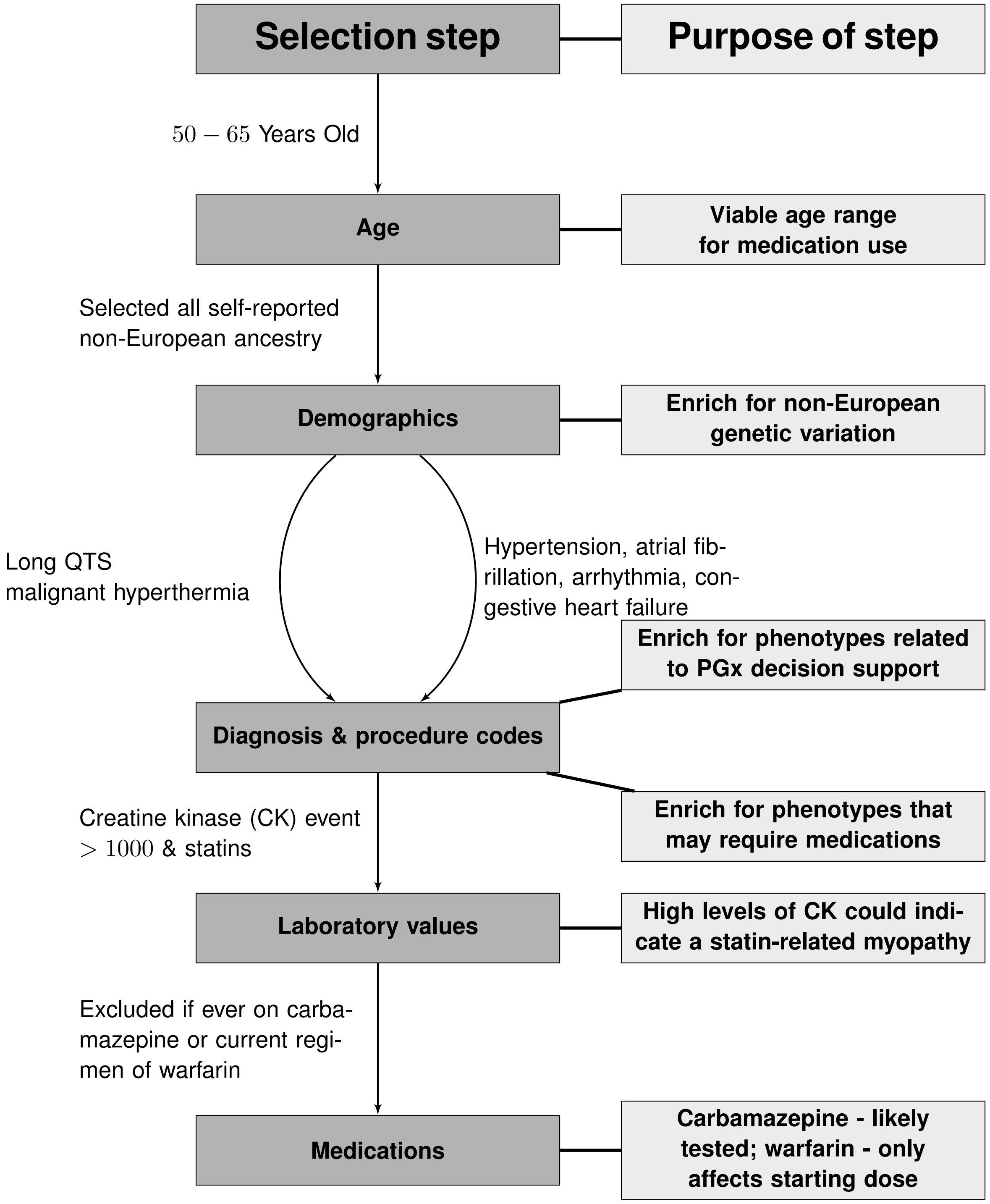

Additional file 1: Figure S1: Selection algorithm for the prospective participant selection and ranking to maximize actionable pharmacogenetic variants and discovery in the eMERGE Network.
