# Supplementary material for: Prospective participant selection and ranking to maximize actionable pharmacogenetic variants and discovery in the eMERGE Network
Source: Genome Med. 2015 Jul 3;7(1):67. doi: 10.1186/s13073-015-0181-z (PMC4517371; doi:10.1186/s13073-015-0181-z)
Supplement: Additional file 2 — Figure S2. Ranking algorithm for the prospective participant selection and ranking to maximize actionable pharmacogenetic variants and discovery in the eMERGE Network. (PDF 73.0KB) [file 13073_2015_181_MOESM2_ESM.pdf]

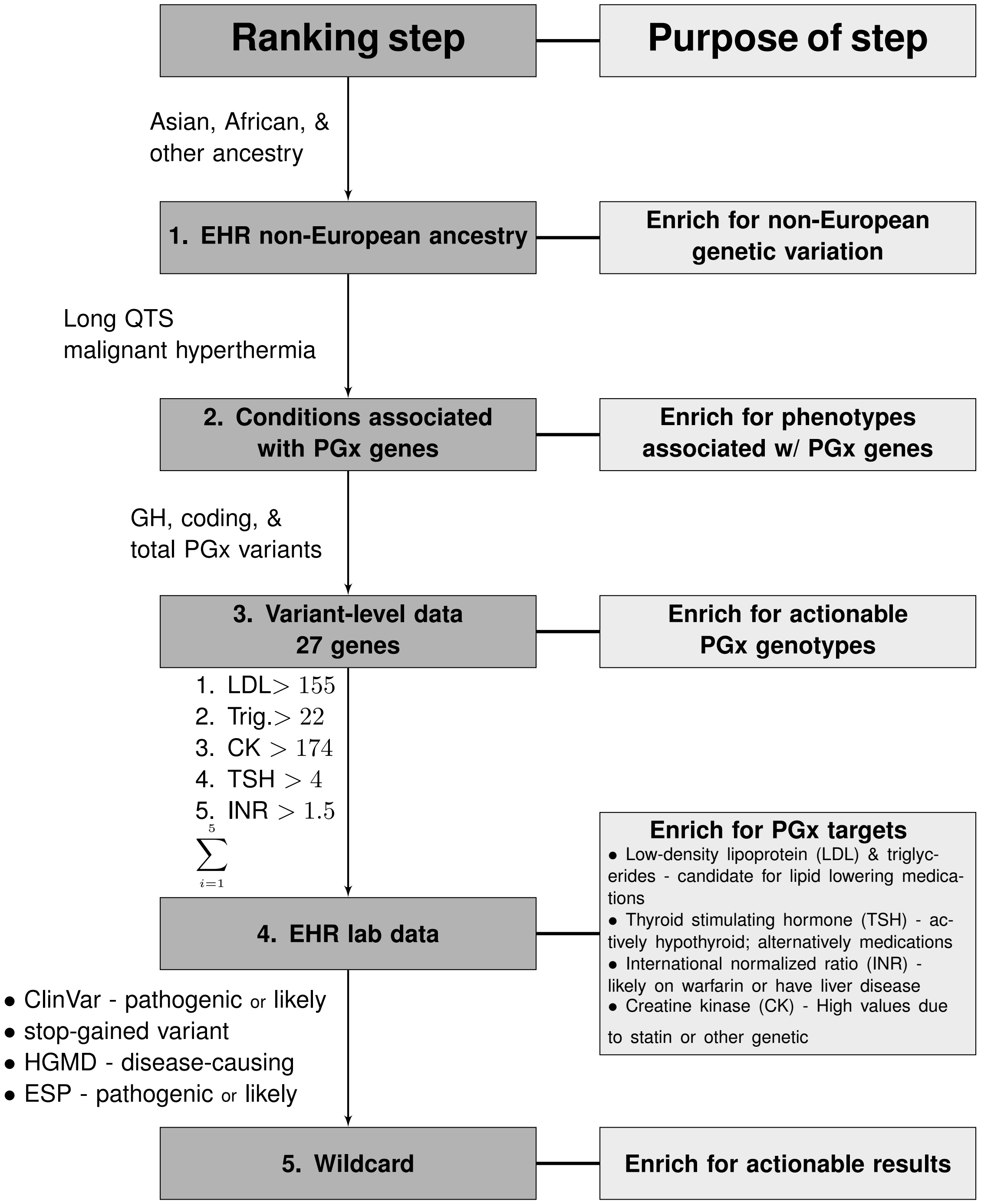

Additional file 2: Figure S2: Ranking algorithm for the prospective participant selection and ranking to maximize actionable pharmacogenetic variants and discovery in the eMERGE Network.
