# Supplementary material for: Prospective participant selection and ranking to maximize actionable pharmacogenetic variants and discovery in the eMERGE Network
Source: Genome Med. 2015 Jul 3;7(1):67. doi: 10.1186/s13073-015-0181-z (PMC4517371; doi:10.1186/s13073-015-0181-z)
Supplement: Additional file 3 — Figure S3. System desgin for the prospective participant selection and ranking to maximize actionable pharmacogenetic variants and discovery in the eMERGE Network; †=IUPAC notation. (PDF 94.5KB) [file 13073_2015_181_MOESM3_ESM.pdf]

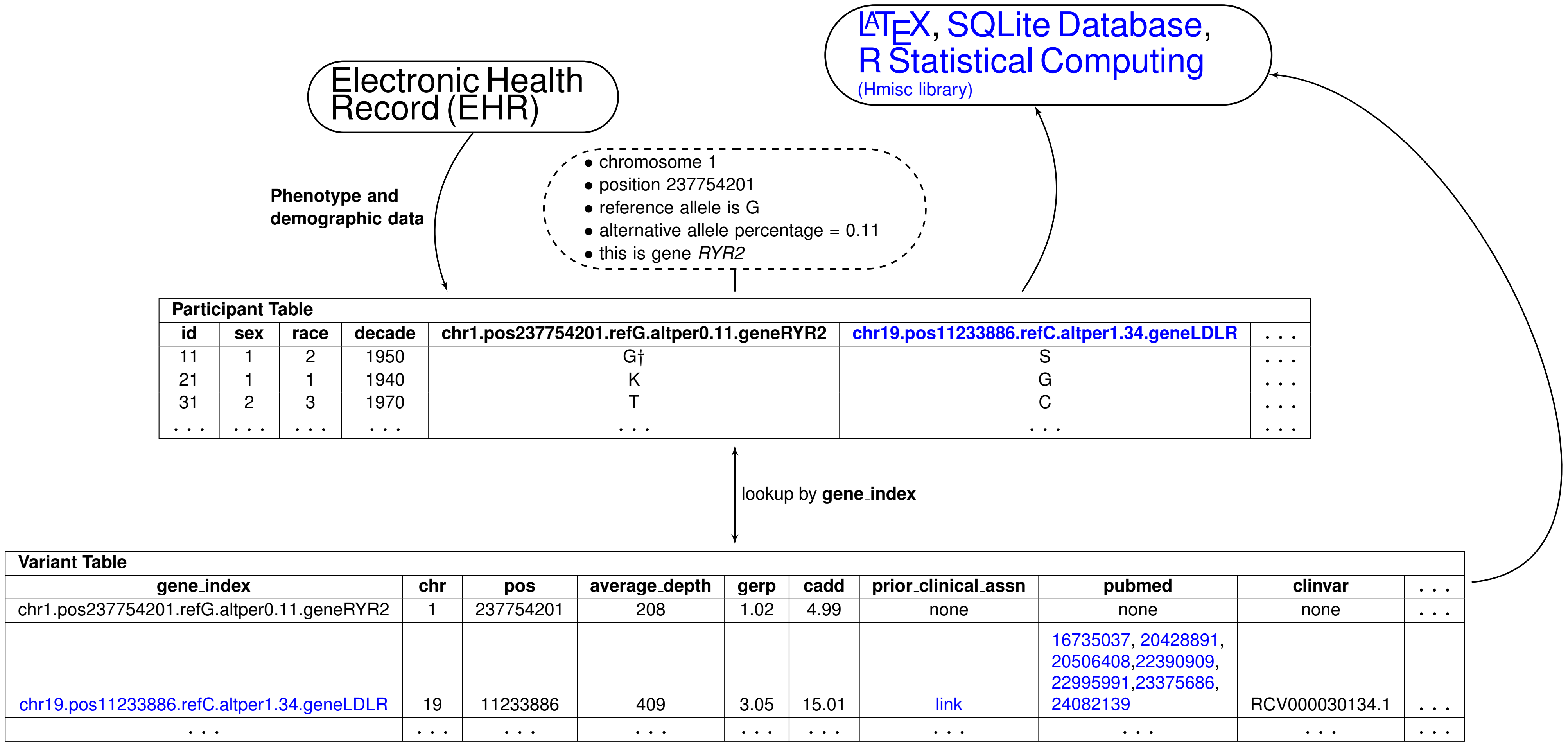

Participant Table

id	sex	race	decade	chr1.pos237754201.refG.altper0.11.geneRYR2	chr19.pos11233886.refC.altper1.34.geneLDLR	. . .
11	1	2	1950	G†	S	. . .
21	1	1	1940	K	G	. . .
31	2	3	1970	T	C	. . .
. . .	. . .	. . .	. . .	. . .	. . .	. . .

Variant Table

gene_index	chr	pos	average_depth	gerp	cadd	prior_clinical_assn	pubmed	clinvar	. . .
chr1.pos237754201.refG.altper0.11.geneRYR2	1	237754201	208	1.02	4.99	none	none	none	. . .
chr19.pos11233886.refC.altper1.34.geneLDLR	19	11233886	409	3.05	15.01	link	16735037, 20428891, 20506408,22390909, 22995991,23375686, 24082139	RCV000030134.1	. . .
. . .	. . .	. . .	. . .	. . .	. . .	. . .	. . .	. . .	. . .

Additional file 3: Figure S3: System desgin for the prospective participant selection and ranking to maximize actionable pharmacogenetic variants and discovery in the eMERGE Network; †=IUPAC notation.

Descriptive Statistics by chr19.pos11233886.refC.altper1.34.geneLDLR

	N	C<br>$N = 882$	Y<br>$N = 12$	Combined<br>$N = 894$	Test Statistic
race	894				$\chi^2_5 = 3.5,\ P = 0.62^1$
american indian or alaska native		1% (11)	0% (0)	1% (11)	
asian		8% (69)	0% (0)	8% (69)	
black or african american		5% (40)	8% (1)	5% (41)	
native hawaiian or other pacific islander		0% (2)	0% (0)	0% (2)	
unknown		2% (19)	8% (1)	2% (20)	
white		84% (741)	83% (10)	84% (751)	
ethnicity	894				$\chi^2_2 = 2.8,\ P = 0.24^1$
N		95% (835)	92% (11)	95% (846)	
U		2% (17)	8% (1)	2% (18)	
Y		3% (30)	0% (0)	3% (30)	
sex	894				$\chi^2_1 = 0.04,\ P = 0.84^1$
F		61% (539)	58% (7)	61% (546)	
M		39% (343)	42% (5)	39% (348)	
hypertension	894				$\chi^2_1 = 0.13,\ P = 0.72^1$
no		21% (184)	17% (2)	21% (186)	
yes		79% (698)	83% (10)	79% (708)	
atrial fibrillation	894				$\chi^2_1 = 0.02,\ P = 0.89^1$
no		90% (798)	92% (11)	90% (809)	
yes		10% (84)	8% (1)	10% (85)	
congestive heart failure	894				$\chi^2_1 = 0.25,\ P = 0.62^1$
no		98% (864)	100% (12)	98% (876)	
yes		2% (18)	0% (0)	2% (18)	
arrhythmia	894				$\chi^2_1 = 0.31,\ P = 0.58^1$
no		50% (439)	42% (5)	50% (444)	
yes		50% (443)	58% (7)	50% (450)	
statins ever	894				$\chi^2_1 = 1.4,\ P = 0.23^1$
FALSE		58% (509)	75% (9)	58% (518)	
TRUE		42% (373)	25% (3)	42% (376)	
warfarin ever	894				$\chi^2_1 = 0.43,\ P = 0.51^1$
FALSE		89% (787)	83% (10)	89% (797)	
TRUE		11% (95)	17% (2)	11% (97)	
smoking status	894				$\chi^2_3 = 0.58,\ P = 0.9^1$
Current Smoke		2% (22)	0% (0)	2% (22)	
Never Smoker		58% (513)	67% (8)	58% (521)	
Past Smoker		39% (344)	33% (4)	39% (348)	
Unknown		0% (3)	0% (0)	0% (3)	
max bmi	893	26.4 **30.4** 36.7 (32.6± 8.5)	25.0 **28.4** 30.5 (29.2± 6.4)	26.4 **30.3** 36.7 (32.5± 8.4)	$F_{1,891} = 2.1,\ P = 0.14^2$
min bmi	893	21.9 **25.0** 29.0 (26.0± 6.0)	20.5 **22.2** 27.6 (22.3± 8.4)	21.9 **25.0** 29.0 (25.9± 6.1)	$F_{1,891} = 2.1,\ P = 0.14^2$
bmi range	893	2.9 **4.8** 8.0 (6.6±5.7)	3.0 **4.2** 6.5 (6.8±7.7)	2.9 **4.8** 8.0 (6.6±5.7)	$F_{1,891} = 0.16,\ P = 0.69^2$
median bmi	893	25 **28** 34 (30± 7)	23 **26** 29 (27± 5)	25 **28** 33 (30± 7)	$F_{1,891} = 2.2,\ P = 0.14^2$
max ldl	741	120 **145** 174 (148± 41)	109 **128** 154 (137± 37)	118 **145** 174 (147± 41)	$F_{1,739} = 0.84,\ P = 0.36^2$
min ldl	741	71 **92** 115 (94± 32)	76 **109** 137 (105± 36)	71 **93** 115 (94± 32)	$F_{1,739} = 0.89,\ P = 0.35^2$
ldl range	741	14 **49** 85 (54±44)	0 **27** 39 (32±44)	14 **49** 85 (54±44)	$F_{1,739} = 3.1,\ P = 0.076^2$
median ldl	741	93 **114** 133 (115± 30)	90 **120** 137 (119± 32)	92 **115** 133 (115± 30)	$F_{1,739} = 0.14,\ P = 0.71^2$
max hdl	891	55 **67** 80 (69±19)	53 **68** 72 (64±12)	55 **67** 79 (69±19)	$F_{1,889} = 0.38,\ P = 0.54^2$
min hdl	891	37 **46** 56 (48±15)	33 **44** 54 (44±17)	37 **46** 56 (48±15)	$F_{1,889} = 0.6,\ P = 0.44^2$
hdl range	891	13 **19** 27 (21±12)	13 **18** 26 (20±12)	13 **19** 27 (21±12)	$F_{1,889} = 0.16,\ P = 0.69^2$
median hdl	891	47 **56** 67 (58±16)	49 **54** 60 (53±13)	47 **56** 67 (58±16)	$F_{1,889} = 0.62,\ P = 0.43^2$
max cholesterol	891	212 **240** 273 (244± 47)	192 **228** 253 (228± 39)	212 **239** 273 (243± 47)	$F_{1,889} = 1.4,\ P = 0.24^2$
min cholesterol	891	148 **170** 190 (170± 33)	134 **170** 193 (166± 40)	148 **170** 190 (170± 33)	$F_{1,889} = 0.07,\ P = 0.79^2$
cholesterol range	891	40 **66** 102 (74± 48)	32 **48** 90 (63± 51)	40 **66** 102 (73± 48)	$F_{1,889} = 0.77,\ P = 0.38^2$
median cholesterol	891	182 **204** 224 (204± 32)	165 **208** 224 (200± 31)	182 **204** 224 (204± 32)	$F_{1,889} = 0.03,\ P = 0.85^2$
max triglyceride	747	106 **166** 254 (234±314)	78 **113** 322 (202±155)	106 **166** 255 (234±312)	$F_{1,745} = 0.22,\ P = 0.64^2$
min triglyceride	747	63 **86** 117 (97± 51)	66 **78** 108 (101± 73)	63 **86** 116 (97± 51)	$F_{1,745} = 0.11,\ P = 0.74^2$
triglyceride range	747	25 **78** 147 (137±302)	0 **36** 181 (101±139)	25 **78** 147 (137±300)	$F_{1,745} = 0.98,\ P = 0.32^2$
median triglyceride	747	86 **119** 167 (138± 84)	78 **113** 152 (130± 81)	86 **119** 167 (138± 84)	$F_{1,745} = 0.18,\ P = 0.68^2$

$a\ b\ c$ represent the lower quartile a, the median b, and the upper quartile c for continuous variables. $x \pm s$ represents $\bar{X} \pm 1$ SD.
N is the number of non–missing values.
Numbers after percents are frequencies.
Tests used:
[1]Pearson test; [2]Wilcoxon test
