# Supplementary material for: Prospective participant selection and ranking to maximize actionable pharmacogenetic variants and discovery in the eMERGE Network
Source: Genome Med. 2015 Jul 3;7(1):67. doi: 10.1186/s13073-015-0181-z (PMC4517371; doi:10.1186/s13073-015-0181-z)
Supplement: Additional file 4 — Table S1. IUPAC Ambiguity Codes; Table S2. PGx Gene List. (PDF 74.9KB) [file 13073_2015_181_MOESM4_ESM.pdf]

Additional file 4: Table S1: IUPAC Ambiguity Codes[?]

Table 1 Additional file 4: Table S1: IUPAC Ambiguity Codes

IUPAC Code	Meaning	Complement
A	A	T
C	C	G
G	G	C
T/U	T	A
M	A or C	K
R	A or G	Y
W	A or T	W
S	C or G	S
Y	C or T	R
K	G or T	M
V	A or C or G	B
H	A or C or T	D
D	A or G or T	H
B	C or G or T	V
N	G or A or T or C	N

Additional file 4: Table S2— Supplemental Table 2: PGx Gene List

Additional file 4: Table S2 is a table listing the 27 actionable genes deemed as either actionable by Clinical Pharmacogenetics Implementation Consortium[?], or as important drug targets based on preliminary association data to pharmacological traits[?].

Table 2 Additional file 4: Table S2: PGx Gene List

GENE	GENE NAME	DRUG/PHENOTYPE
ABCA1	ATP-binding cassette transporter	Multidrug Resistance
ABCB1	ATP-binding cassette, sub-family B (MDR/TAP), member 1	Multidrug Resistance
APOA1	Apolipoprotein A-I	HDL
APOB	Apolipoprotein B	LDL
CACNA1S	Calcium channel, voltage-dependent, L type, alpha 1S subunit	Malignant Hyperthermia
CFTR	Cystic fibrosis transmembrane conductance regulator	Ivacaftor
CYP2C9	Cytochrome P450 2C9	Warfarin
CYP2C19	Cytochrome P450 2C19	Clopidogrel, TCAs
CYP2D6	Cytochrome P450 2D6	Codeine, SSRIs, TCAs
CYP3A4	Cytochrome P450 3A4	Tacrolimus
CYP3A5	Cytochrome P450 3A5	Tacrolimus
CYP3A7	Cytochrome P450 3A7	Tacrolimus
DPYD	Dihydropyrimidine dehydrogenase	5FU/Capecitabine
KCNH2	Potassium voltage-gated channel, subfamily H, member 2	Arrhythmia
G6PD	Glucose-6-phosphate dehydrogenase	Rasburicase, Septra
HLA–B	Major histocompatibility complex, class I, B	Abacavir, Allopurinol, Carbamazepine, Phenytoin
HMGCR	HMG-CoA reductase	Cholesterol
IFNL3	Interferon, lambda 3	Pegintron
LDLR	Low density lipoprotein receptor	Cholesterol
NAT2	N-acetyltransferase 2 (arylamine N-acetyltransferase)	Arylamine, Hydrazine
RYR1	Ryanodine receptor 1	Malignant hyperthermia
RYR2	Ryanodine receptor 2	Arrhythmia
SCN5A	Sodium channel, voltage-gated, type V, alpha subunit	Arrhythmia
SLCO1B1	Solute carrier organic anion transporter family member 1B1	Simvastatin
TPMT	Thiopurine methyltransferase	Thiopurines
UGT1A1	UDP-glucuronosyltransferase 1-1	Irinotecan
VKORC1	Vitamin K epoxide reductase complex subunit 1	Warfarin
